# Anaplasmosis in a 73-Year-Old Male From South Florida: A Case Report

**DOI:** 10.7759/cureus.77767

**Published:** 2025-01-21

**Authors:** Jatin Goyal, Alexandra Goldman, Camille C Go

**Affiliations:** 1 Internal Medicine, Herbert Wertheim College of Medicine, Florida International University, Miami, USA; 2 Family Medicine, Baptist Health of South Florida, Miami, USA

**Keywords:** anaplasma phagocytophilum, fever, human granulocytic anaplasmosis, ixodes scapularis, pancytopenia, tick-borne illness

## Abstract

Anaplasmosis is a tick-borne illness transmitted by the *Ixodes scapularis* tick in the Northeast and Midwest regions of the United States. Clinical symptoms of anaplasmosis can be non-specific, which may delay the diagnosis. This is a case of a 73-year-old male from South Florida who initially presented with non-specific febrile illness to an urgent care and was initially treated for a viral infection. Persistent febrile episodes prompted presentation to the emergency room. Initial findings of pancytopenia and fever worsened after the initiation of broad-spectrum antibiotics. Upon further history, the patient recounted recent travel to upstate New York and Canada, prompting a switch to intravenous doxycycline therapy for the presumptive diagnosis of tick-borne disease. Definitive diagnosis of anaplasmosis was confirmed via polymerase chain reaction. Since discharge with doxycycline therapy, the patient’s symptoms and pancytopenia have fully resolved. Conducting a complete history and physical examination with concurrent laboratory studies is imperative for accurate diagnoses and improvement in patient outcomes.

## Introduction

From 2020 to 2022, there has been a significant increase in the number of tick-borne illnesses. The largest number was reported in 2022, with 71,346 cases of tick-borne illnesses [1]. Of these, Lyme disease was the most common. However, another condition that can present similarly to Lyme disease is human granulocytic anaplasmosis (HGA or anaplasmosis) due to overlapping clinical features. HGA is caused by the *Anaplasma phagocytophilum* organism, an obligate intracellular gram-negative bacterium [2]. This bacterium is most commonly transmitted by two different ticks, *Ixodes scapularis*, which is predominately found in the Northeast and Midwest regions, and *Ixodes pacificus*, which is predominately found on the western coast of the United States [1,3]. HGA is most prevalent during the summer months, aligning with peak tick activity. While the ticks that transmit *Anaplasma phagocytophilum* are typically associated with the Northwestern regions, 5,651 cases of HGA have been reported nationwide. In Florida, specifically, the incidence of HGA in 2022 was recorded at 1.24 cases per million [4].

Other tick-borne illnesses include spotted fever rickettsioses, ehrlichiosis (*Ehrlichia chaffeensis *or *Ehrlichia ewingii*), babesiosis, tularemia, Powassan virus disease, and undetermined ehrlichiosis/anaplasmosis [1]. Lyme disease and babesiosis are common co-infections with anaplasmosis due to transmission by the same tick. While tick bites are the most common form of transmission of HGA, rare cases of blood transfusions may lead to illness [5].

Clinical symptoms of anaplasmosis infection typically occur one to two weeks after the initial tick bite. The most common symptoms are fever, headache, malaise, myalgia, and arthralgia. Common laboratory findings are elevated alanine transaminases (ALT) or aspartate transaminases (AST), thrombocytopenia, and leukopenia. Diagnosis is typically made through a thorough history and physical examination, as well as travel history. Confirmatory test is done via multiple methods including serology testing, immunohistochemistry staining for the bacterium, polymerase chain reaction (PCR) of DNA, and isolation of culture. PCR of DNA is regarded as one of the quicker confirmatory tests with a sensitivity of approximately 67%-90% and a specificity of 60%-85% [5]. Here we present a case of a 73-year-old male from South Florida who initially presented with an acute febrile illness and was diagnosed with anaplasmosis, a condition uncommon in this region and therefore often overlooked as a result.

## Case presentation

A 73-year-old male from South Florida with a past medical history of hypertension and hyperlipidemia initially experienced an acute onset of fever, chills, body aches, and mild nasal congestion in early July. On the second day of symptoms, he sought care at an urgent care clinic, reporting fevers ranging from 100°F to 102°F, accompanied by chills, lethargy, mild shortness of breath, headaches, and nausea. He denied dysuria, diarrhea, or vomiting. At the urgent care clinic, a rapid COVID-19 test was performed, which returned negative. The patient was advised to continue supportive care with symptomatic management. At this time, the patient's wife, who had been experiencing similar flu-like symptoms, was recovering, yet the patient reported that his condition continued to worsen over the following two days. He experienced increasing weakness and a maximum fever of 105°F that was unresponsive to over-the-counter acetaminophen, which ultimately prompted him to seek care at the emergency department (ED) in Miami. In the ED, the patient appeared acutely ill and febrile, with his main concern being muscle weakness. His vital signs were remarkable for a temperature of 101.6°F, blood pressure of 134/76 mmHg, heart rate of 85 beats per minute, respiratory rate of 16 breaths per minute, and an oxygen saturation of 96%. All other review of systems and physical examination findings were unremarkable. Laboratory workup was significant for leukopenia (2.6 x 10^9^/L), thrombocytopenia (57 x 10^9^/L), hyponatremia (131 mEq/L), hypokalemia (2.6 mEq/L), and hypocalcemia (7.5 mg/dL) (additional laboratory values are given in Table 1). Given the patient's status, he was admitted and started on broad-spectrum intravenous (IV) antibiotics, vancomycin and piperacillin-tazobactam.

**Table 1:** Laboratory results during hospitalization BUN, blood urea nitrogen; eGFR, estimated glomerular filtration rate; LDH, lactate dehydrogenase; TIBC, total iron-binding capacity

Laboratory Tests	Hospital Day				
	1	2	3	4	5
Blood count					
Hemoglobin (g/dL)	14.5	12.7	12.1	12.6	13
Hematocrit (%)	41.3	36.1	35	36.4	38.3
White blood cell (k/µL)	2.62	1.74	1.51	2.36	3.54
Platelet count (k/µL)	57	31	25	31	61
Differential type					
Neutrophils (%)	88.1	-	48.9	45.8	-
Immature granulocytes (%)	0.4	-	0.7	0.4	-
Lymphocytes (%)	6.1	-	35.8	40.3	-
Monocytes (%)	5	-	13.9	11.4	-
Eosinophils (%)	0	-	0	1.3	-
Basophils (%)	0.4	-	0.7	0.8	-
Absolute neutrophils (k/µL)	2.31	-	0.74	1.08	-
Chemistry					
Sodium (mEq/L)	131	133	135	138	138
Potassium (mEq/L)	2.6	2.9	3.3	3.6	3.6
Chloride (mEq/L)	97	100	104	107	105
Bicarbonate (mEq/L)	25	26	26	27	28
Anion gap (mEq/L)	9	7	5	4	5
Glucose (mg/dL)	148	129	98	102	97
Creatinine (mg/dL)	0.95	0.87	0.68	0.64	0.67
BUN (mg/dL)	16	15	14	10	11
BUN/creatinine ratio	16.8	17.2	20.6	15.6	16.4
eGFR (mL/min)	85	>90	>90	>90	>90
Calcium (mg/dL)	7.5	7.2	7.9	7.8	8.4
Haptoglobin	-	-	113	-	-
Ferritin (ng/mL)	1,918	-	4,550	-	1,557
Iron (mcg/dL)	-	-	36	-	-
TIBC (mcg/dL)	-	-	202	-	-
LDH (U/L)	-	296	386	423	341
Cytokine receptor					
IL-2 receptor alpha (U/mL)	-	-	-	1,391	-

On day two, the patient continued to have febrile episodes, temperature of 105°F, and was now noted to have worsening pancytopenia. Infectious disease and hematology were consulted. Upon further investigation, the patient disclosed that he and his wife returned from a trip to New York and Canada a little over a week ago, during which they spent one afternoon hiking through a forested meadow. The patient recalled that both he and his wife were wearing long pants and closed toe shoes to protect themselves from the semi-tall grass. With this new information, a microbiology serology panel (Table 2) was ordered to determine the causative organism. The patient was also switched to IV doxycycline, 100 mg twice a day, due to suspicion of a tick-borne illness given his recent travel history. Additional workup included testing for anemia, hemolysis, hepatitis infection, and human immunodeficiency virus (HIV) infection.

**Table 2:** Infectious pathogens tested during hospitalization HGE, human granulocytic ehrlichiosis; RSV, respiratory syncytial virus

Diagnostic Tests	Result
Hepatitis B surface antigen	Non-reactive
Hepatitis B surface antibody	<3.1
Hepatitis B core ABS-total	Non-reactive
Hepatitis C antibody	Non-reactive
Dengue fever IgG	0.09
Dengue fever IgM	0.61
Cytomegalovirus IgG	Positive
Cytomegalovirus IgM	Negative
PARV-parvovirus B19 Ab IgG	6.8
PARV-parvovirus B19 Ab IgM	0.1
*Streptococcus pneumoniae *Ab specimen	Urine
*Streptococcus pneumoniae* antigen	Negative
*Legionella pneumophilia *Ag	Negative
HIV 1 and 2 antibodies	Non-reactive
*Ehrlichia chaffeensis*, PCR	Negative
*Anaplasma phagocytophilum*, PCR	Positive
*Erhlichia chaffeensis* IgG	Negative
*Erhlichia chaffeensis *IgM	Negative
HGE IgG titer	Negative
HGE IgM titer	Negative
Influenza A by PCR	Not detected
Influenza B by PCR	Not detected
Parainfluenza 1 by PCR	Not detected
Parainfluenza 2 by PCR	Not detected
Parainfluenza 3 by PCR	Not detected
Parainfluenza 4 by PCR	Not detected
Metapneumovirus by PCR	Not detected
Adenovirus by PCR	Not detected
Coronavirus 229E by PCR (not COVID-19)	Not detected
Coronavirus HKU1 by PCR (not COVID-19)	Not detected
Coronavirus NL63 by PCR (not COVID-19)	Not detected
Coronavirus OC43 by PCR (not COVID-19)	Not detected
Rhinovirus/enterovirus by PCR	Not detected
RSV by PCR	Not detected
*Bordetella pertussis* by PCR	Not detected
*Bordetella parapertussis* by PCR	Not detected
*Chlamydia pneumoniae* by PCR	Not detected
*Mycoplasma pneumoniae* by PCR	Not detected
SARS-CoV-2 (COVID-19)	Not detected

On the third day of hospital admission and after 24 hours of doxycycline treatment, the patient was now afebrile. He continued to deny abdominal pain, nausea, vomiting, diarrhea, and bleeding. However, the patient’s inflammatory markers, specifically lactate dehydrogenase, continued to trend upward. Given the increased severity of pancytopenia, the patient underwent a bone marrow biopsy due to concern regarding myelodysplastic syndromes. By the fourth day of the hospital admission, the patient expressed decreased fatigue and weakness and now had been afebrile for 48 hours. The bone marrow report indicated monoclonal plasma cells and an atypical myeloid maturation process, a finding that seemed unrelated to the current clinical presentation. However, despite symptomatic improvement, the patient’s vitals and laboratory results still suggested an ongoing inflammatory condition. Given the lack of findings related to nutritional deficiency or hemolysis from previous tests, the prolonged febrile episode, and elevated ferritin levels, hemophagocytic lymphohistiocytosis (HLH) was added to the differential diagnosis as a possible cause of the persistent inflammation. This prompted further evaluation by testing for soluble IL-2 receptor alpha chains (Table 1). On the patient’s fifth and final day in the hospital, the patient had been afebrile for 72 hours with continued improvement of his initial symptoms, especially his levels of fatigue and weakness. Though the patient had an elevated soluble IL-2 receptor alpha chain level, the upward trend in his hemoglobin, white blood cells, and platelet counts helped the medical team rule down the diagnosis of HLH and reinforced the suspicion of an infectious process. As such, the patient was cleared for discharge with doxycycline (100 mg tablet twice a day) for 10 days.

The patient’s ongoing improvement with doxycycline treatment suggested a tick-borne illness, which was confirmed by PCR testing, identifying *Anaplasma phagocytophilum* as the causative organism of HGA. The patient has since returned to full recovery and was further instructed to follow up with his primary care provider for repeat labs to fully assess his return to complete recovery.

## Discussion

HGA is a tick-borne illness caused by the bacterium *Anaplasma phagocytophilum*, primarily found in the northeastern United States [1]. A systematic review of 110 patients with anaplasmosis identified that the most common mode of transmission is through tick vectors. The study found that fever is a prominent symptom, occurring in 90% of patients, while 76% also experienced thrombocytopenia [6]. The clinical features of our case - severe fever, thrombocytopenia, and leukopenia - closely align with the typical presentation of anaplasmosis.

Tick-borne illnesses can often go overlooked due to their lower incidence compared to viral infections. Lyme disease is frequently the first tick-borne illness considered, while anaplasmosis, ehrlichiosis, and babesiosis are less commonly examined. *Anaplasma phagocytophilum* has an incubation period of one to two weeks and can present with symptoms ranging from mild to severe, including end organ damage [5]. In this case, the absence of a rash helped to rule out Lyme disease, while the patient’s rapid improvement with doxycycline therapy made a diagnosis of babesiosis less likely [5,7].

Accurate diagnosis of anaplasmosis relies heavily on clinical evaluation, including a detailed history of potential tick exposure and travel to endemic areas. Clinical suspicion can be confirmed with PCR, serology, and blood smears. The treatment for anaplasmosis is doxycycline with a dosage of 100 mg by mouth or intravenously twice a day for 10 days in adults [7].

The incidence of anaplasmosis in Florida is notably low, recorded at only 1.24 cases per million in 2022 [4]. This rarity contributes to the condition being frequently overlooked in clinical practice, as it is not commonly considered a primary diagnosis for febrile illnesses in this region. In our case, HGA was initially overlooked as a potential diagnosis, partly due to an incomplete history of present illness that did not fully capture the patient’s travel history, and partly because of its low incidence in Florida. Consequently, this condition was not considered by multiple healthcare professionals, resulting in a delay in initiating appropriate treatment.

Delays in treatment can lead to complications and increase morbidity and mortality rates. Such delays can significantly increase the risk of complications, including seizures, coma, major organ failure (renal, respiratory, or cardiac), and sepsis. Moreover, patients may be more vulnerable to co-infections with opportunistic pathogens such as *Aspergillus* spp., herpes simplex virus, and *Candida* spp. An additional complication includes HLH, an immune-mediated diffuse destruction of tissue [5,8].

This case highlights the importance of maintaining a high index of suspicion for HGA, regardless of the region of the United States in which the patient is presenting. Even in areas like South Florida, where the incidence is exceedingly low, clinicians should consider anaplasmosis in the differential diagnosis of febrile illnesses, particularly when there is a history of travel to endemic regions. Early recognition and timely treatment not only mitigate severe complications but also lead to improved patient outcomes.

## Conclusions

Tick-borne illnesses such as anaplasmosis, though uncommon, should not be overlooked as the cause of classic symptoms on first presentation. A thorough workup that includes a complete history, physical, and laboratory studies is imperative for accurate diagnosis. Early recognition enables prompt treatment, which not only leads to a reduction in minor consequences, such as length of hospital stay, fever, and flu-like symptoms, but also reduces the risk for serious complications including opportunistic infections, organ failure, and death.
